# Inferring About the Average Value of Audit Errors from Sequential Ratio Tests

**DOI:** 10.3390/e26110998

**Published:** 2024-11-20

**Authors:** Grzegorz Sitek, Mariusz Pleszczyński

**Affiliations:** 1 Department of Statistics, Econometrics and Mathematics, University of Economics in Katowice, ul. 1 Maja 50, 40-287 Katowice, Poland; grzegorz.sitek@ue.katowice.pl; 2 Department of Mathematics Applications and Methods for Artificial Intelligence, Faculty of Applied Mathematics, Silesian University of Technology, 44-100 Gliwice, Poland

**Keywords:** sequential likelihood ratio test, mixture of probability distribution, Monte Carlo method

## Abstract

The book amounts are modeled as values of a random variable, represented by a mixture of distributions of both the correct and error-contaminated amounts. The mixing coefficient represents the proportion of items with non-zero error amounts. This study addresses the problem of determining the sample size needed for testing statistical hypotheses regarding mean accounting errors. The average sample size is estimated using the Sequential Probability Ratio Test (SPRT), applying the Monte Carlo method. Estimating average audit errors is a common challenge in economic research.

## 1. Introduction

Let $X_{i}$ represent the book amount of the ith item in an account, with the total population book amount denoted as $X_{U}=\sum_{i=1}^{N}x_{i}$. At regular intervals, an auditor samples *n* line items from the account and to compare against their correct values. Therefore, let $Y_{i}$ denote the audited amount for the ith line item, and let $\tau_{i}=X_{i}-Y_{i}$ denote the error amount. Note that the total book amount is known to the auditor. The fundamental issue is constructing confidence limits for means or totals in finite populations where the underlying distribution is highly skewed and contains a substantial proportion of zero values. This situation is often encountered in statistical applications such as statistical auditing, reliability assessments, and insurance. The most distinctive feature of accounting data is the large proportion of line items without error, while an audit sample may not yield any nonzero error amounts. For data with mostly zero observations, classical interval estimation based on asymptotic normality is not reliable. In auditing practice, auditors are often more interested in obtaining lower or upper confidence limits than in obtaining two-sided confidence intervals. Specifically, independent public accountants are focused on estimating the lower confidence bound for the total audited amount to avoid overestimation and potential legal liability. Stringer and Kaplan (see [1,2]) have demonstrated that accounting populations are highly positively skewed, with considerable diversity in the characteristics of error amounts across subsystems. There are several distributions that also exhibit the same form of the distribution observed in accounting populations. These include the Gamma, Log-normal, Weibull, and Beta distributions. The error rates are usually very low, which render many existing statistical procedures inappropriate for estimating and hypothesis testing of error rates and error amounts. There are two main types of audit tests where statistical sampling is advantageous. The first is a compliance test, used to determine the procedural error rate in a population of transactions. The second is a substantive test of details, aimed at evaluating the aggregate monetary error in a stated balance. Inferences about the total error amount are usually made using confidence intervals, which are closely related to hypothesis testing. The decision-making process in auditing is treated as a problem of testing statistical hypotheses about admissibility of the total or the mean accounting errors. This approach allows auditors to control both the significance level (risk of incorrect rejection) and the type II error probability (risk of incorrect acceptance). Substantive tests of details verify the accuracy of recorded monetary values in financial statements, providing direct evidence regarding the accuracy of the total recorded values. Auditors may apply substantive tests extensively or use compliance tests to evaluate the efficiency and effectiveness of internal controls in mitigating material errors. In compliance tests, the variable of interest is the error rate (proportion of transactions for which the internal control operates wrongly). Samples of transactions are used to infer this error rate.

## 2. A Mixture of Probability Distributions as a Model for Generating Accounting Values

Wywiał in [3] proposed the following model. Let *U* be the population of accounting documents of size *N* with given accounting totals. Some of the documents contain errors. In the population *U*, there are given accounting totals (values) $x_{i}$ for each element i∈U. Let $x^{T}=\left[x_{1},\cdots ,x_{N}\right]\in R_{+}^{N}$ be the observation of a random vector $X^{T}=\left[X_{1},\cdots ,X_{N}\right]$. We denote the true book values (without errors) by $y_{i}$, i∈U and let $y^{T}=\left[y_{1},\cdots ,y_{N}\right]\in R_{+}^{N}$ be the random vector observation $Y^{T}=\left[Y_{1},\cdots ,Y_{N}\right]$. Vector of accounting values contaminated by errors $w^{T}=\left[w_{1},\cdots ,w_{N}\right]$ will be an observation of the random vector $W^{T}=\left[W_{1},\cdots ,W_{N}\right]$. Finally, let $Z^{T}=\left[Z_{1},\cdots ,Z_{N}\right]$, where $Z_{i}=0$$\left(Z_{i}=1\right)$ if $X_{i}=Y_{i}$$\left(X_{i}\neq Y_{i}\right)$.

In practice, all values of *X* are known before auditing process. Observations *x* of *X* are treated as a specific auxiliary data. Auditing process leads to observation of values $Z_{i}$, $Y_{i}$ and $W_{i}$, i∈U. Let $\bar{X}=\frac{1}{N}\sum_{i\in U}X_{i}$, $\bar{Y}=\frac{1}{N}\sum_{i\in U}Y_{i}$, $\bar{W}=\frac{1}{N}\sum_{i\in U}W_{i}$. Their values will be denoted $\bar{x}$, $\bar{y}$, $\bar{w}$.

Let an auditor arbitrarily select a sample *s* of size *n* from *U*. Hence, $X_{s}$ is the subvector of *X*, n≤N. The random vector $X_{s}$ is observed in *s* where the objects are controlled. After the auditing process, the sample *s* is split into two disjoint sub-samples $s_{0}$ and $s_{1}$ where $s=s_{0}\cup s_{1}$. The set $s_{1}$ is of size $n_{1}=k$ and the set $s_{0}$ is of size $n_{0}=n-k$. In the sub-sample $s_{0}$, there are observed accounting amounts without errors. Before the auditing process, we have observations of the following data:

$$X=\left(X_{i}:i\in U\right)=\left(X_{s},X_{U-s}\right),$$

where

$$X_{s}=\left(X_{i}:i\in s\right),\;X_{U-s}=\left(X_{i}:i\in U-s\right).$$

After the auditing process, we have observations of the following data:

$$T_{U}=\left(T_{s},X_{U-s}\right),\;T_{s}=\left((X_{i},Z_{i}):i\in s\right)=\left(Y_{s_{0}},W_{s_{1}}\right).$$

Values *T*, $T_{U}$, *X*, $X_{s}$, $X_{U-s}$, $Y_{s_{0}}$ and $W_{s_{1}}$ are denoted, respectively, by *t*, $t_{U}$, *x*, $x_{s}$, $x_{U-s}$, $y_{s_{0}}$ and $w_{s_{1}}$. In the following work, we assume that $y_{s_{0}}=y_{s}$ and $w_{s_{1}}=w_{s}$.

Let $\tau =E(\bar{X}-\bar{Y})$ be the expected mean accounting error. Audit purpose is inference on τ or on the expected total accounting error $N\tau =E(\sum_{i\in U}X_{i}-\sum_{i\in U}Y_{i})$.

Let $F_{0}\left(y|\theta_{0}\right)$ be the probability distribution function of the random variable *Y*, whose values are true accounting and $\theta_{0}\in \Theta_{0}$ where $\Theta_{0}$ is the parameter space. The distribution function of *W* is denoted by $F_{1}\left(w|\theta_{1}\right)$, where $\theta_{1}\in \Theta_{1}$. Moreover, let $\Theta =\Theta_{0}\cup \Theta_{1}$. We assume that an accounting errors appears with probability *p*. We can write Z=1 when an accounting error occurs P(Z=1)=p and Z=0 when it does not occur P(Z=0)=1−p. According to the well-known total probability theorem we have: $F\left(x\right)=F\left(x|Z=0\right)P\left(Z=0\right)+F\left(x|Z=1\right)P\left(Z=1\right)$ and finally

(1) $$F\left(x|\theta \right)=\left(1-p\right)F_{0}\left(x|\theta_{0}\right)+\;\;pF_{1}\left(x|\theta_{1}\right),$$

where $\theta =\theta_{0}\cup \theta_{1}$ and $\theta \in \Theta =\Theta_{0}\cup \Theta_{1}$ is the parameter space. Hence, the probability distribution of the observed accounting amounts is a mixture of the distribution function $F_{0}\left(x\right|\theta_{0}$) of the true amounts and the distribution function $F_{1}\left(x\right|\theta_{1}$) of the amounts contaminated by errors. When the random variables *Y* and *W* are continuous, by differentiating both sides of Equation (1) we have

(2) $$f\left(x|\theta \right)=\left(1-p\right)f_{0}\left(x|\theta_{0}\right)+\;\;pf_{1}\left(x|\theta_{1}\right).$$

Therefore, the probability density of the observed accounting amounts is a mixture of density $f_{0}\left(x\right|\theta_{0}$) of the true amounts and density $f_{1}\left(x\right|\theta_{1}$) of the amounts contaminated by errors. Let *R* and *Y* be independent and *R* is the accounting error. Hence W=Y+R, X=Y+ZR, X=(1−Z)Y+ZW. The basic moments of the random variable *X* are:

(3) E(X)=(1−p)E(X|Z=0)+pE(X|Z=1)=(1−p)E(Y)+pE(W),

(4) $$V\left(X\right)=p\left(1-p\right)(E\left(W\right)-E\left(Y\right){)}^{2}+pV\left(W\right)+\left(1-p\right)V\left(Y\right).$$

### 2.1. A Mixture of Gamma Probability Distributions as a Model for Generating Accounting Values

The well-known gamma probability distribution we denote by G(α,β), where parameters α>0 and β>0 are called scale and shape parameters. The shape of gamma density distribution does not depend on the scale parameter because its skewness and kurtosis coefficients are equal to $\frac{2}{\sqrt{\beta }}$ and $\frac{6}{\beta }$, respectively. Wywiał in [3] considered the model based on a mixture of gamma distributions. Let Y∼G(a,c) and R∼G(b,c) be independent random variables. The advantage of this model is that the density function for the sum of gamma distributions can be determined. Based on the above assumption, the random variable W=Y+R∼G(a+b,c). Using the previous considerations, we obtain

(5) $$f\left(x|a,b,c\right)=\left(1-p\right)f_{0}\left(x|a,c\right)+\;\;pf_{1}\left(x|a,b,c\right),$$

where

(6) $$f_{1}\left(x|a,b,c\right)=\frac{c^{a+b}}{\Gamma (a+b)}x^{a+b-1}e^{-cx},\;x>0,$$

(7) $$f_{0}\left(x|a,c\right)=\frac{c^{a}}{\Gamma (a)}x^{a-1}e^{-cx},\;x>0.$$

From Formulas (3) and (4) we obtain

(8) $$E\left(X\right)=\frac{a+pb}{c},\;\;V\left(X\right)=\frac{a+pb+p\left(1-p\right)b^{2}}{c^{2}}.$$

For more on the use of gamma decomposition to model accounting values, see the articles [4,5]. The book amounts are treated as values of a random variable which distribution is a mixture of the distributions of correct amount and the distribution of the true amount contaminated by error. Distributions of correct amount and true amount contaminated by error are right-skewed because small book amounts are more frequent than large book amounts. It is convenient to assume that the book values are additive function of true accounting amounts and accounting errors. Hence, we can expect that the above proposed quite simple model describes accounting data well.

### 2.2. A Mixture of Poisson Probability Distributions as a Model for Generating Accounting Values

We assume that the variable Y∼Pois(a) (true values) and R∼Pois(b) (accounting error), where Pois(λ) is a Poisson distribution with probability function

(9) $$P\left(k\right)=\frac{\lambda^{k}e^{-\lambda }}{k!}\;\mathrm{for}\;k=0,1,2,\cdots$$

If variables *Y* and *R* are independent, then the random variable W=Y+R∼Pois(a+b).

(10) $$P\left(x\right)=\left(1-p\right)P_{0}\left(x|a\right)+pP_{1}\left(x|a,b\right),$$

(11) $$\begin{array}{c} P_{1}\left(x|a,b\right)=\frac{{\left(a+b\right)}^{x}e^{-a-b}}{x!},\;\;x=0,1,2,\ldots ,\\ P_{0}\left(x|a\right)=\frac{{\left(a\right)}^{x}e^{-a}}{x!},\;\;x=0,1,2.\ldots \end{array}$$

From Formulas (3) and (4) we obtain

(12) $$E\left(X\right)=\left(1-p\right)a+p\left(a+b\right)=a+pb,\;\;V\left(X\right)=a+bp\left(b+1-pb\right).$$

## 3. Sequential Tests Based on the Ratio of the Likelihood Function

The set of issues involved in proceeding sequentially in verifying statistical hypotheses is called sequential analysis (see [6]). Sequential analysis was created by Wald (see [7]). The sequential approach to statistical inference was the subject of systematic research during the Second World War. This research was concerned with the quality of munitions and was conducted by the Statistical Research Group at Columbia University. Data in sequential analysis are used both to decide when to end an observation (data collection) and to draw actual conclusions (regarding the parameter being estimated or hypotheses being tested). The sequential method is applicable in auditing when assessing the internal control system in compliance testing when verifying hypotheses regarding the proportion of accounting errors. In their doctoral thesis, Byekwaso in [8] used a polynomial Dirichlet model to estimate and test hypotheses about error rates in accounting populations using Bayesian methods with sequential stopping rules.

### 3.1. Sequential Ratio Test

Let f(x,θ) be the density (probability) function with θ=[τ,γ], where τ is subject to verification, and γ is the set of parameters indifferent from the point of view of hypothesis testing $H_{0}\left(\tau =\tau_{0}\right)$ against $H_{1}\left(\tau =\tau_{1}\right)$. We propose a hypothesis $H_{0}(\theta =\theta_{0})$. Hypothesis $H_{0}$ is verified by comparing with the Hypothesis $H_{1}\left(\theta =\theta_{1}\right)$, where $\theta_{1}$ is some specific value different from $\theta_{0}$, and $\theta_{j}=\left[\tau_{j},\gamma \right]$, j=0,1. During the Sequential Probability Ratio Test (SPRT), we progressively sample one or more items at each stage of the sequential hypothesis verification. Depending on the predetermined errors of the α and β, the numbers $A=\frac{1-\beta }{\alpha }$ and $B=\frac{\beta }{1-\alpha }$ satisfying the condition 0<B<1<A. The elements are drawn into a simple sample. For the first drawn observation $x_{1}$, let us determine the value of the ratio $\frac{f(x_{1},\theta_{1})}{f(x_{1},\theta_{0})}$. If

- $\frac{f(x_{1},\theta_{1})}{f(x_{1},\theta_{0})}\geq A$ then we reject the Hypothesis $H_{0}$ in favor of the hypothesis $H_{1}$.
- $\frac{f(x_{1},\theta_{1})}{f(x_{1},\theta_{0})}\leq B$ then we accept the Hypothesis $H_{0}$.
- $B<\frac{f(x_{1},\theta_{1})}{f(x_{1},\theta_{0})}<A$ then we select the next element to sample.

In sequential proceedings, it is more convenient to operate with logarithms of numbers *A* and *B* and random variables

(13) $$z_{i}=\ln\frac{f(x_{i},\theta_{1})}{f(x_{i},\theta_{0})},\;\;i=1,2,\ldots ,n.$$

If in the sample we have *m* elements (m≥1) then if

- $\sum_{i=1}^{m}z_{i}\geq \ln A$ then we reject the Hypothesis $H_{0}$ in favour of the hypothesis $H_{1}$;
- $\sum_{i=1}^{m}z_{i}\leq \ln B$ then we accept the Hypothesis $H_{0}$;
- $\ln B<\sum_{i=1}^{m}z_{i}<\ln A$ then we select the next element to sample.

Let *X* be a random variable with parameter θ and of density f(x,θ). We consider the following simple hypotheses about the value of the parameter θ

(14) $$H_{0}:\theta =\theta_{0},\;\;H_{1}:\theta =\theta_{1}>\theta_{0}.$$

The verification of the null hypothesis (14) consists of calculating on the basis of an *n* elementary random sample $\left(X_{1},\ldots ,X_{n}\right)$ the value of the statistic:

(15) $$l_{n}=\ln\frac{f(X_{1},\ldots ,X_{n},\theta_{1})}{f(X_{1},\ldots ,X_{n},\theta_{0})}=\sum_{i=1}^{n}\ln\frac{f(x_{i},\theta_{1})}{f(x_{i},\theta_{0})},$$

and comparing it with the designated constants *A* and *B*. The SPRT test can be extended to versions in which the unknown parameters are replaced by their maximum-likelihood estimators ([9]) and then the previously described sequential procedure is applied

(16) $$l_{n}=\sum_{i=1}^{n}\ln\frac{f(x_{i},{\hat{\theta }}_{1})}{f(x_{i},{\hat{\theta }}_{0})},$$

(17) $$\hat{\theta_{j}}=\underset{\theta_{j}\in \Theta_{j}}{\arg\;\;\max}\prod_{i=1}^{n}f\left(x_{i},\theta_{j}\right),\;\;j=0,1,$$

where $\Theta_{j}=\left[\tau_{j},\gamma \right]$, $\hat{\theta_{j}}=\left[\tau_{j},\hat{\gamma }\right]$.

### 3.2. The Expected Sample Size

The approximate expected value of the sample size for a sequential ratio test is given by the formula ([6]):

(18) $$E_{\theta }\left(n\right)=\frac{L(\theta )\ln B+(1-L(\theta ))\ln A}{E_{\theta }\left(z\right)},$$

where: $z=\ln\frac{f(x,\theta_{1})}{f(x,\theta_{0})}$, L(θ) is the OC function of the sequential ratio test. The OC function describes the probability of accepting the Hypothesis $H_{0}$ ([10]). The functions L(θ) are written with the formula

(19) $$L\left(\theta \right)=\frac{A^{h_{0}\left(\theta \right)}-1}{A^{h_{0}\left(\theta \right)}-B^{h_{0}\left(\theta \right)}}.$$

Function $h_{0}\left(\theta \right)$ for a variable of *x* of continuous type satisfies the following condition

(20) $$\int_{-\infty }^{\infty }{\left(\frac{f(x,\theta_{1})}{f(x,\theta_{0})}\right)}^{h_{0}\left(\theta \right)}f\left(x,\theta \right)dx=1.$$

Function $h_{0}\left(\theta \right)$ for a variable of *x* of discrete type satisfies the following condition

(21) $$\sum_{j}{\left(\frac{f(x_{j},\theta_{1})}{f(x_{j},\theta_{0})}\right)}^{h_{0}\left(\theta \right)}f\left(x_{j},\theta \right)=1.$$

The expected value E(n) is determined from the formula

(22) $$E\left(n\right)=\frac{1}{2}\left(\frac{(1-\alpha )\ln B+\alpha \ln A}{E_{\alpha }\left(n\right)}+\frac{\beta \ln B+(1-\beta )\ln A}{E_{\beta }\left(n\right)}\right).$$

We use the above expressions to determine the expected sample size for tests on the mean value of accounting errors. We test the hypothesis

(23) $$H_{0}:\;\tau =\tau_{0},\;\;H_{1}:\tau =\tau_{1}>\tau_{0},$$

where τ denotes the mean value of the accounting errors determined from the mixture of Poisson or gamma distribution.

The expected value $E_{\alpha }\left(n\right)$ is determined by the formula:

(24) $$E_{\alpha }\left(n\right)=\sum_{x=0}^{\infty }P_{0}\left(X=x\right)\ln\frac{P_{1}\left(X=x\right)}{P_{0}\left(X=x\right)},$$

where:

(25) $$P_{1}\left(X=x\right)=\frac{e^{-a}\left(1-p\right)a^{x}}{x!}+\frac{pe^{-a-\frac{\tau_{1}}{p}}{\left(a+\frac{\tau_{1}}{p}\right)}^{x}}{x!},$$

and

(26) $$P_{0}\left(X=x\right)=\frac{e^{-a}\left(1-p\right)a^{x}}{x!}+\frac{pe^{-a-\frac{\tau_{0}}{p}}{\left(a+\frac{\tau_{0}}{p}\right)}^{x}}{x!}.$$

The expected value $E_{\beta }\left(n\right)$ is determined by the formula:

(27) $$E_{\beta }\left(n\right)=\sum_{x=0}^{\infty }P_{1}\left(X=x\right)\ln\frac{P_{1}\left(X=x\right)}{P_{0}\left(X=x\right)}.$$

The expected value $E_{\alpha }\left(n\right)$ for a mixture of gamma distributions is determined from the formula:

(28) $$E_{\alpha }\left(n\right)=\int_{0}^{\infty }g_{0}\left(x\right)\ln\frac{g_{1}\left(x\right)}{g_{0}\left(x\right)}dx.$$

The expected value $E_{\beta }\left(n\right)$ is determined from the formula:

(29) $$E_{\beta }\left(n\right)=\int_{0}^{\infty }g_{1}\left(x\right)\ln\frac{g_{1}\left(x\right)}{g_{0}\left(x\right)}dx,$$

where:

(30) $$g_{0}\left(x\right)=\frac{\left(1-p\right)c^{a}e^{-cx}x^{a-1}}{\Gamma (a)}+\frac{pc^{a+(\frac{c\tau_{0}}{p})}e^{-cx}x^{a+(\frac{c\tau_{0}}{p})-1}}{\Gamma \left(a+\frac{c\tau_{0}}{p}\right)},$$

and

(31) $$g_{1}\left(x\right)=\frac{\left(1-p\right)c^{a}e^{-cx}x^{a-1}}{\Gamma (a)}+\frac{pc^{a+(\frac{c\tau_{1}}{p})}e^{-cx}x^{a+(\frac{c\tau_{1}}{p})-1}}{\Gamma \left(a+\frac{c\tau_{1}}{p}\right)}.$$

For fixed parameters *a*, *c*, $\tau_{i}$, *p* expected values $E_{\alpha }\left(n\right)$ i $E_{\beta }\left(n\right)$ can be calculated by suitable numerical integration methods using, for example, Mathematica.

### 3.3. Simulation-Based Sample Size Determination

The purpose of the simulation study was to determine the average sample size $\bar{n}$ and simulation probabilities of accepting and rejecting the Hypothesis $H_{0}$. The determination of the average sample size using the Monte Carlo method was dealt with by Boiroju (see [11]). The purpose of an audit may be to certify the accuracy of a financial settlement, in which case a situation arises where the auditor focuses more on minimizing risk β rather than α. Therefore, $\bar{n}$ was estimated under the assumption that the observed data in the sample were generated under the assumption that the Hypothesis $H_{1}$ is true.

1. For fixed values of α and β we determine $a_{g}=\ln\frac{1-\beta }{\alpha }$ and $b_{g}=\ln\frac{\beta }{1-\alpha }$. To the variables LP, *j*, *k* are assigned the values LP=1000, j=k=n=0.
2. For i=1,2,…,LP we generate a dataset of size N=4000 from a mixture of Poisson distributions $\frac{e^{-a}\left(1-p\right)a^{x}}{x!}+\frac{pe^{-a-b}{\left(a+b\right)}^{x}}{x!}$ with the following parameters: a=1000, p=0.1. The parameter *b* is determined from the formula $b=\frac{\tau_{1}}{p}$.
3. We draw a sample size $n_{0}=0.01N$, $n=n+n_{0}$. We divide the population into subsets $y_{s}$, $w_{s}$, $x_{U-s}$.
4. Estimate the mixture parameters $a_{1}$ and $p_{1}$ provided $b=\frac{\tau_{1}}{p}$ based on the credibility function. For the parameters obtained, we calculate the logarithm of the credibility function $l_{1}$.
5. Estimate the mixture parameters $a_{0}$ and $p_{0}$ provided $b=\frac{\tau_{0}}{p}$ based on the credibility function. For the parameters obtained, we calculate the logarithm of the credibility function $l_{0}$.
6. Let us calculate the value of the test statistic $S_{i}=l_{1}-l_{0}$.
7. Repeat steps 3 to 6 until $b_{g}<S_{i}<a_{g}$ and n≤0.82N.
8. If $S_{i}\geq a_{g}$ then k=k+1, which results in the rejection of the Hypothesis $H_{0}$ in favour of the Hypothesis $H_{1}$.
9. If $S_{i}\leq b_{g}$ then j=j+1, which means that we reject the Hypothesis $H_{1}$ even though the hypothesis is true.
10. For each *i* we determine the number $n_{i}$ of sample elements necessary to decide whether to accept or reject the hypothesis $H_{0}$. We stop the algorithm when $n_{i}=0.82N$.
11. Determine the mean sample size $\bar{n}=\frac{1}{LP}\sum_{i=1}^{LP}n_{i}$, the standard deviation of the simulated sample sizes $S\left(\bar{n}\right)=\sqrt{\frac{1}{LP-1}\sum_{i=1}^{LP}{\left(n_{i}-\bar{n}\right)}^{2}}$, the simulated probability of accepting the Hypothesis $H_{0}:$$\hat{\beta }=\frac{j}{LP}$, simulation probability of rejecting the Hypothesis $H_{0}:$$\hat{m}=1-\hat{\beta }=\frac{k}{LP}$ and simulation probability $\hat{\kappa }=\hat{m}+\hat{\beta }$ of terminating a sequential procedure below a threshold n≤0.82N.

For a mixture of Poisson distributions, the following hypothesis was tested:

$$H_{0}:\;\tau =\tau_{0}=5,\;\;H_{1}:\tau =\tau_{1}>\tau_{0}.$$

**Figure 1:**
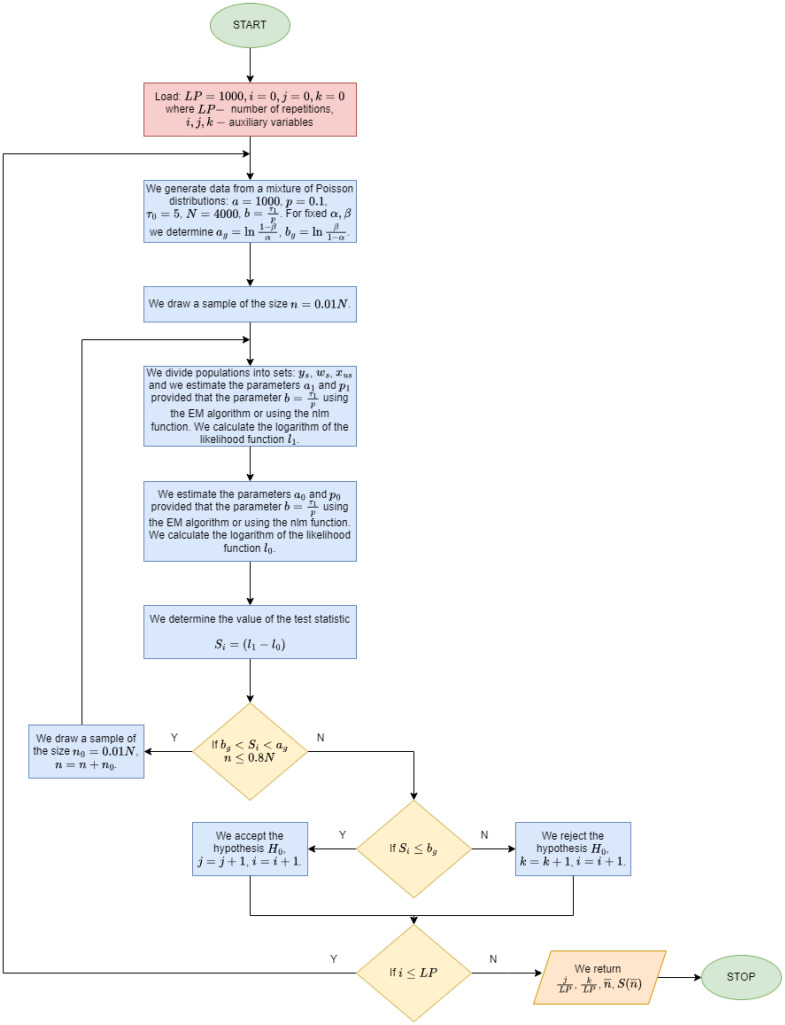
Algorithm for simulation-based determination of sample sizes and hypothesis rejection probabilities.

Details of how the algorithm works are shown in Figure 1.

The nlm function uses the Newton–Raphson algorithm and is included in the R package. Simulations were performed for three mixing values p=0.1, p=0.2, p=0.3 for a mixture of Poisson distribution. The corresponding parameters were determined for the mixing parameter $b=\frac{\tau_{1}}{p}$.

Table 1, Table 2, Table 3, Table 4, Table 5 and Table 6 show the results $E_{\beta }\left(n\right)$ obtained from Formulas (24)–(27) for a mixture of Poisson distributions. The values of $E_{\beta }\left(n\right)$ have been determined in Mathematica. For a mixture of gamma distributions, the following hypothesis was tested:

$$H_{0}:\;\tau =\tau_{0}=50,\;\;H_{1}:\tau =\tau_{1}>\tau_{0}.$$

Parameters were used to generate a mixture of gamma distributions: a=2, c=0.002. For the mixing parameters p=0.1, p=0.2, p=0.3 the corresponding parameters were determined $b=\frac{c\tau_{1}}{p}$. The results of the simulation studies are presented in Table 7, Table 8, Table 9, Table 10, Table 11 and Table 12.

The average sample size decreases with increasing $\tau_{1}$. The smallest sample sizes are obtained for data from a mixture of Poisson distributions generated with the parameter p=0.1. For α=β=0.2 and for $\tau_{1}=1.3\tau_{0}$ the mean sample value is 149 (3.7% of the population size). For this parameter, the arithmetic mean of the contributing error values $\bar{w}=a+b$ is the largest. Similarly for p=0.2 the mean sample value is 307 (7.7% of the population size) and for the p=0.3 the mean sample value is 502 (12.6% of the population size).

The sample mean values obtained are lower than the values obtained using Formulas (24)–(27). Comparing values E(n) and $\bar{n}$ in Table 1, Table 2, Table 3, Table 4, Table 5 and Table 6 only makes sense if the value of $\hat{\kappa }$ is close to unity. This means that only then have almost all iterations of simulated sample sizes needed finished before the population size is exhausted. For a fixed value of α, changing the value of β causes negligible changes in the mean of sample size. The simulation probability $\hat{\beta }$ of accepting the Hypothesis $H_{0}$ in Table 1, Table 2, Table 3, Table 4, Table 5 and Table 6 is smaller than assumed. Reasons for this may be that the critical values of the Wald test are approximated by ($a_{g},b_{g}$) or the method of drawing, in which we draw 1%N− population elements rather than individual elements, i.e., in our case there is a sequential draw of 40 population elements.

**Figure 2:**
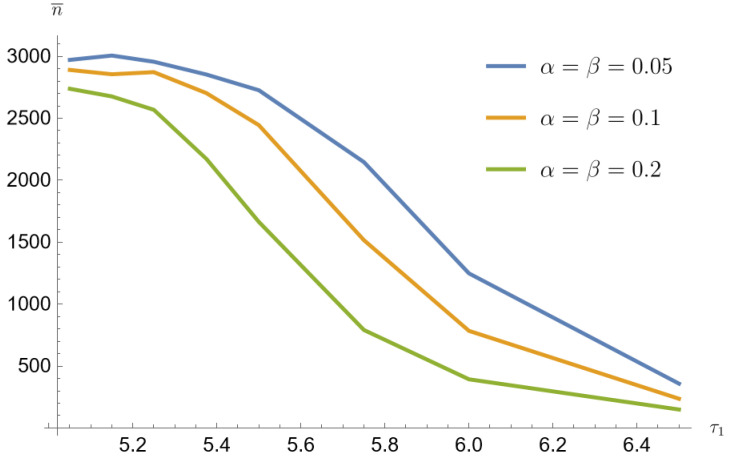
Simulated sample sizes-mixture of Poisson distributions p=0.1, $\tau_{0}=5$ (based on Table 1 and Table 2).

**Figure 3:**
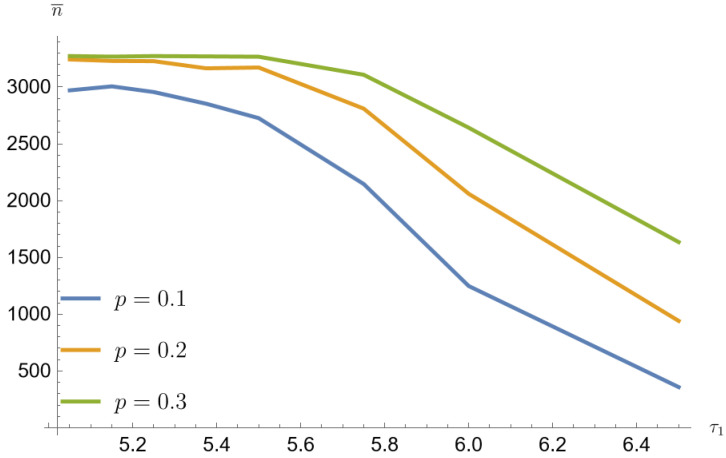
Simulated sample sizes for different parameter mixing-mixture Poisson distributions for α=β=0.05, $\tau_{0}=5$ (based on Table 1 and Table 6).

**Figure 4:**
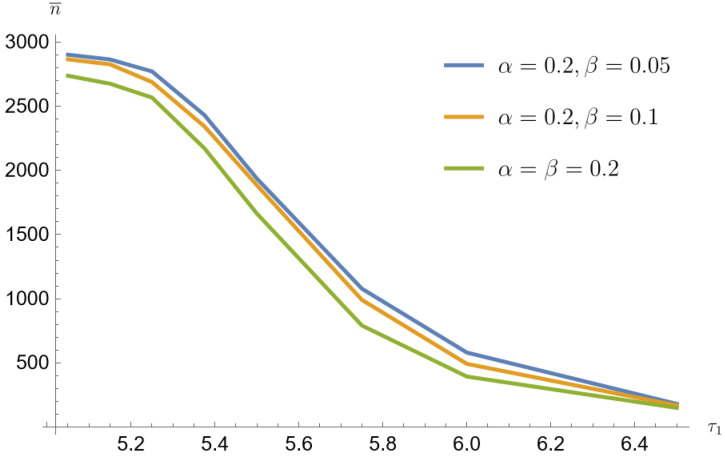
Simulated sample sizes with fixed α and variable β-mixture of Poisson distributions for p=0.1, $\tau_{0}=5$ (based on Table 1 and Table 2).

**Figure 5:**
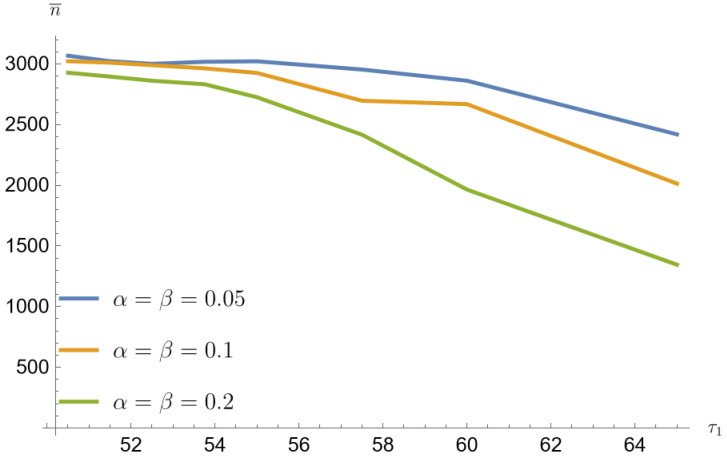
Simulated sample sizes-mixture of gamma distributions for p=0.1, $\tau_{0}=50$ (based on Table 7 and Table 8).

**Figure 6:**
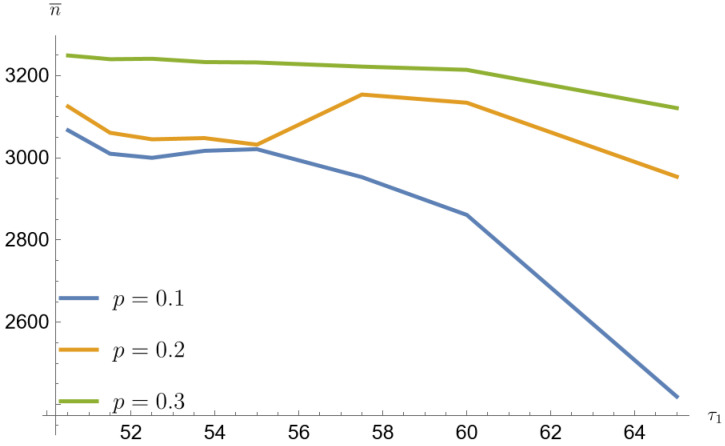
Simulated sample sizes for different parameter mixing-mixture gamma distributions for α=β=0.05, $\tau_{0}=50$ (based on Table 7, Table 8, Table 9, Table 10, Table 11 and Table 12).

Figure 1, Figure 2, Figure 3, Figure 4, Figure 5 and Figure 6 show that the average sample size decreases with increasing $\tau_{1}$. The smallest sample sizes are obtained for data from a mixture of gamma distributions generated with the parameter p=0.1. For α=β=0.2 and for $\tau_{1}=1.3\tau_{0}$ the average sample size is 1345 (33.7% population size). For this parameter, the arithmetic mean of the contributing error values $\bar{w}=\frac{a+b}{c}$ is the largest. Similarly for p=0.2 sample mean value is 1848 (46.2% population size), and for p=0.3 average sample value is 2280 (57% population size). For p=0.1, α=β=0.05 and for $\tau_{1}=1.3\tau_{0}$ the average sample value is 2419 (60.5% population size). Similarly for p=0.2 average sample size is 2954 (73.9% population size), and for p=0.3 average sample size is 3121 (78% population size).

## 4. Conclusions

This paper presents the application of a sequential test based on the likelihood ratio function for audit studies. The main objective of the simulation studies was to determine the expected sample size.

Section 3.2 outlines formulas for numerically calculating the expected sample size when testing the hypothesis of average accounting errors using a mixture of Poisson distributions as the underlying model for accounting values. The expected sample sizes for the mixture of Poisson distributions were obtained both analytically and through simulation. For the mixture of gamma distributions, the expected sample sizes values were determined exclusively through simulation.

The values of $E_{\beta }\left(n\right)$ obtained in Table 1, Table 2, Table 3, Table 4, Table 5 and Table 6 indicate that this sequential test is practical when the mean error size $\tau_{1}$ significantly exceeds $\tau_{0}$. Otherwise, the expected sample size $E_{\beta }\left(n\right)$ becomes impractically large compared to the typical population size of accounting documents. The simulation studies were conducted only for selected hypothetical and alternative parameters (average audit errors), due to the time-consuming nature of the simulations. It is possible that different parameter settings could yield more favorable outcomes in terms of efficiency.

## Figures and Tables

**Table 1:** Sequential test results. Mixture of Poisson distributions p=0.1, $\tau_{0}=5$.

$\tau_{1}$	α	β	$\bar{n}$	$E_{\beta }\left(n\right)$	$S(\bar{n})$	$\hat{\beta }$	$\hat{\kappa }$
1.01$\tau_{0}$	0.05	0.05	2971	528,571	808	0.069	0.139
	0.05	0.1	2987	473,960	780	0.057	0.141
	0.05	0.2	2922	380,261	825	0.12	0.186
	0.1	0.05	2961	473,960	795	0.046	0.156
	0.1	0.1	2890	350,608	862	0.104	0.191
	0.1	0.2	2827	271,813	911	0.128	0.223
	0.2	0.05	2902	267,598	857	0.069	0.185
	0.2	0.1	2866	228,528	868	0.085	0.216
	0.2	0.2	2738	165,907	970	0.122	0.278
1.03$\tau_{0}$	0.05	0.05	3006	57,867	738	0.059	0.139
	0.05	0.1	2887	18,410	885	0.104	0.185
	0.05	0.2	2867	41,630	882	0.143	0.209
	0.1	0.05	2914	51,888	856	0.063	0.175
	0.1	0.1	2855	38,384	901	0.095	0.211
	0.1	0.2	2732	29,757	975	0.159	0.276
	0.2	0.05	2865	29,296	870	0.075	0.213
	0.2	0.1	2827	25,018	890	0.094	0.238
	0.2	0.2	2676	18,163	992	0.16	0.322
1.05$\tau_{0}$	0.05	0.05	2956	20,531	808	0.059	0.155
	0.05	0.1	2923	18,410	836	0.097	0.181
	0.05	0.2	2759	14,770	941	0.155	0.275
	0.1	0.05	2814	18,410	920	0.078	0.23
	0.1	0.1	2872	13,618	855	0.091	0.22
	0.1	0.2	2715	10,558	992	0.144	0.28
	0.2	0.05	2772	10,394	925	0.076	0.276
	0.2	0.1	2689	8876	969	0.094	0.323
	0.2	0.2	2568	6444	1020	0.175	0.391
1.075$\tau_{0}$	0.05	0.05	2853	8963	905	0.073	0.204
	0.05	0.1	2813	8037	918	0.105	0.233
	0.05	0.2	2737	6448	947	0.151	0.289
	0.1	0.05	2794	8037	915	0.065	0.268
	0.1	0.1	2703	5945	979	0.093	0.301
	0.1	0.2	2539	4609	1065	0.157	0.381
	0.2	0.05	2431	4537	1094	0.076	0.445
	0.2	0.1	2343	3875	1111	0.117	0.492
	0.2	0.2	2172	2813	1129	0.15	0.568

**Table 2:** Sequential test results. Mixture of Poisson distributions p=0.1, $\tau_{0}=5$.

$\tau_{1}$	α	β	$\bar{n}$	$E_{\beta }\left(n\right)$	$S(\bar{n})$	$\hat{\beta }$	$\hat{\kappa }$
1.1$\tau_{0}$	0.05	0.05	2726	4954	961	0.089	0.293
	0.05	0.1	2618	4442	1021	0.104	0.354
	0.05	0.2	2518	3564	1090	0.164	0.383
	0.1	0.05	2505	4442	1039	0.072	0.428
	0.1	0.1	2444	3286	1066	0.104	0.466
	0.1	0.2	2241	2547	1125	0.16	0.546
	0.2	0.05	1936	2508	1192	0.076	0.644
	0.2	0.1	1884	2142	1205	0.095	0.661
	0.2	0.2	1663	1555	1195	0.163	0.736
1.15$\tau_{0}$	0.05	0.05	2146	2128	1141	0.065	0.591
	0.05	0.1	2059	1908	1151	0.103	0.624
	0.05	0.2	1869	1531	1188	0.123	0.69
	0.1	0.05	1610	1908	1179	0.063	0.77
	0.1	0.1	1516	1412	1158	0.081	0.786
	0.1	0.2	1351	1094	1154	0.145	0.826
	0.2	0.05	1078	1077	1061	0.063	0.898
	0.2	0.1	991	920	1035	0.06	0.895
	0.2	0.2	791	668	866	0.131	0.953
1.2$\tau_{0}$	0.05	0.05	1248	1159	1088	0.051	0.865
	0.05	0.1	1237	1039	1077	0.081	0.873
	0.05	0.2	1045	834	996	0.102	0.913
	0.1	0.05	896	872	943	0.042	0.939
	0.1	0.1	784	768	875	0.059	0.957
	0.1	0.2	686	596	814	0.11	0.956
	0.2	0.05	580	586	762	0.037	0.966
	0.2	0.1	492	501	687	0.048	0.982
	0.2	0.2	393	363	547	0.106	0.993
1.3$\tau_{0}$	0.05	0.05	358	484	527	0.024	0.996
	0.05	0.1	340	434	509	0.03	0.991
	0.05	0.2	324	348	471	0.05	0.996
	0.1	0.05	268	364	433	0.024	0.997
	0.1	0.1	236	321	352	0.027	0.999
	0.1	0.2	215	249	324	0.044	1
	0.2	0.05	180	245	292	0.022	1
	0.2	0.1	168	209	237	0.033	1
	0.2	0.2	149	152	196	0.05	1

**Table 3:** Sequential test results. Mixture of Poisson distributions p=0.2, $\tau_{0}=5$.

$\tau_{1}$	α	β	$\bar{n}$	$E_{\beta }\left(n\right)$	$S(\bar{n})$	$\hat{\beta }$	$\hat{\kappa }$
1.01$\tau_{0}$	0.05	0.05	3243	1,338,250	258	0.011	0.026
	0.05	0.1	3231	1,199,990	310	0.014	0.031
	0.05	0.2	3226	962,757	290	0.034	0.05
	0.1	0.05	3200	1,007,080	384	0.011	0.05
	0.1	0.1	3235	887,683	279	0.015	0.036
	0.1	0.2	3191	688,186	396	0.033	0.063
	0.2	0.05	3213	677,515	336	0.017	0.047
	0.2	0.1	3195	578,594	391	0.027	0.06
	0.2	0.2	3178	420,049	406	0.04	0.077
1.03$\tau_{0}$	0.05	0.05	3230	147,470	324	0.017	0.032
	0.05	0.1	3196	132,233	429	0.027	0.047
	0.05	0.2	3195	106,092	405	0.041	0.063
	0.1	0.05	3211	110,976	369	0.014	0.043
	0.1	0.1	3205	97,818	393	0.022	0.047
	0.1	0.2	3190	75,835	410	0.038	0.062
	0.2	0.05	3193	74,659	390	0.02	0.064
	0.2	0.1	3195	63,758	393	0.03	0.062
	0.2	0.2	3165	46,287	446	0.037	0.089
1.05$\tau_{0}$	0.05	0.05	3228	52,652	335	0.011	0.028
	0.05	0.1	3199	47,212	390	0.026	0.054
	0.05	0.2	3176	37,878	445	0.052	0.069
	0.1	0.05	3200	39,622	376	0.02	0.056
	0.1	0.1	3207	34,925	394	0.021	0.046
	0.1	0.2	3164	27,076	468	0.057	0.085
	0.2	0.05	3182	26,656	436	0.021	0.072
	0.2	0.1	3187	22,764	404	0.02	0.074
	0.2	0.2	3109	16,526	531	0.062	0.127
1.075$\tau_{0}$	0.05	0.05	3165	23,161	468	0.016	0.074
	0.05	0.1	3192	20,768	401	0.036	0.061
	0.05	0.2	3149	16,662	467	0.065	0.099
	0.1	0.05	3182	17,429	423	0.021	0.073
	0.1	0.1	3181	15,363	427	0.029	0.074
	0.1	0.2	3121	11,910	545	0.07	0.112
	0.2	0.05	3009	11,725	661	0.029	0.199
	0.2	0.1	2996	10,013	675	0.025	0.203
	0.2	0.2	2779	7269	883	0.086	0.317

**Table 4:** Sequential test results. Mixture of Poisson distributions p=0.2, $\tau_{0}=5$.

$\tau_{1}$	α	β	$\bar{n}$	$E_{\beta }\left(n\right)$	$S(\bar{n})$	$\hat{\beta }$	$\hat{\kappa }$
1.1$\tau_{0}$	0.05	0.05	3172	12,895	410	0.024	0.09
	0.05	0.1	3157	11,562	442	0.031	0.095
	0.05	0.2	3050	9277	625	0.1	0.16
	0.1	0.05	3036	9704	632	0.019	0.181
	0.1	0.1	3037	8553	616	0.035	0.181
	0.1	0.2	2878	6631	792	0.118	0.275
	0.2	0.05	2653	6528	978	0.019	0.364
	0.2	0.1	2649	5575	956	0.04	0.394
	0.2	0.2	2314	4048	1125	0.129	0.524
1.15$\tau_{0}$	0.05	0.05	2810	5615	824	0.028	0.321
	0.05	0.1	2750	5035	868	0.059	0.36
	0.05	0.2	2448	4039	1081	0.156	0.472
	0.1	0.05	2379	4225	1084	0.03	0.502
	0.1	0.1	2316	3724	1115	0.048	0.519
	0.1	0.2	1951	2887	1186	0.164	0.67
	0.2	0.05	1790	2842	1228	0.022	0.703
	0.2	0.1	1641	2427	1216	0.056	0.747
	0.2	0.2	1373	1763	1160	0.172	0.827
1.2$\tau_{0}$	0.05	0.05	2060	3095	1136	0.042	0.657
	0.05	0.1	1958	2775	1156	0.067	0.68
	0.05	0.2	1705	2226	1164	0.175	0.764
	0.1	0.05	1670	2329	1208	0.04	0.75
	0.1	0.1	1570	2053	1180	0.076	0.794
	0.1	0.2	1244	1591	1071	0.166	0.887
	0.2	0.05	1176	1567	1117	0.044	0.872
	0.2	0.1	1023	1338	1039	0.082	0.907
	0.2	0.2	783	972	891	0.154	0.949
1.3$\tau_{0}$	0.05	0.05	941	1321	941	0.029	0.945
	0.05	0.1	851	1185	877	0.072	0.959
	0.05	0.2	704	950	770	0.135	0.977
	0.1	0.05	667	994	798	0.032	0.965
	0.1	0.1	632	876	739	0.079	0.978
	0.1	0.2	461	680	584	0.125	0.988
	0.2	0.05	450	669	623	0.034	0.987
	0.2	0.1	377	571	522	0.063	0.994
	0.2	0.2	307	415	391	0.135	0.998

**Table 5:** Sequential test results. Mixture of Poisson distributions p=0.3, $\tau_{0}=5$.

$\tau_{1}$	α	β	$\bar{n}$	$E_{\beta }\left(n\right)$	$S(\bar{n})$	$\hat{\beta }$	$\hat{\kappa }$
1.01$\tau_{0}$	0.05	0.05	3273	1,795,990	115	0.001	0.004
	0.05	0.1	3270	1,610,440	138	0.003	0.006
	0.05	0.2	3264	1,292,060	188	0.004	0.008
	0.1	0.05	3266	1,351,540	164	0.003	0.008
	0.1	0.1	3255	1,191,310	223	0.007	0.014
	0.1	0.2	3256	923,575	223	0.005	0.012
	0.2	0.05	3258	909,255	206	0.006	0.013
	0.2	0.1	3264	776,498	183	0.004	0.008
	0.2	0.2	3262	563,724	182	0.005	0.01
1.03$\tau_{0}$	0.05	0.05	3268	198,788	150	0.005	0.007
	0.05	0.1	3268	178,250	166	0.002	0.006
	0.05	0.2	3256	143,011	224	0.007	0.013
	0.1	0.05	3262	149,595	194	0.003	0.009
	0.1	0.1	3263	131,859	188	0.003	0.009
	0.1	0.2	3264	102,225	184	0.006	0.009
	0.2	0.05	3273	100,640	121	0.002	0.004
	0.2	0.1	3268	85,946	146	0.001	0.007
	0.2	0.2	3256	62,395	206	0.006	0.014
1.05$\tau_{0}$	0.05	0.05	3273	71,288	121	0.001	0.004
	0.05	0.1	3265	63,922	172	0.005	0.008
	0.05	0.2	3258	43,800	208	0.01	0.012
	0.1	0.05	3262	53,646	192	0.004	0.009
	0.1	0.1	3270	47,286	145	0.002	0.006
	0.1	0.2	3267	36,659	152	0.006	0.009
	0.2	0.05	3253	36,090	228	0.005	0.015
	0.2	0.1	3254	30,821	225	0.004	0.016
	0.2	0.2	3241	22,375	256	0.011	0.029
1.075$\tau_{0}$	0.05	0.05	3270	31,530	144	0.003	0.006
	0.05	0.1	3261	28,272	186	0.007	0.012
	0.05	0.2	3253	22,683	226	0.018	0.019
	0.1	0.05	3267	23,727	175	0.003	0.009
	0.1	0.1	3265	20,914	155	0.006	0.013
	0.1	0.2	3247	16,214	241	0.0.012	0.025
	0.2	0.05	3202	15,962	359	0.002	0.06
	0.2	0.1	3210	13,632	316	0.004	0.063
	0.2	0.2	3134	9896	477	0.031	0.114

**Table 6:** Sequential test results. Mixture of Poisson distributions p=0.3, $\tau_{0}=5$.

$\tau_{1}$	α	β	$\bar{n}$	$E_{\beta }\left(n\right)$	$S(\bar{n})$	$\hat{\beta }$	$\hat{\kappa }$
1.1$\tau_{0}$	0.05	0.05	3267	17,650	150	0.003	0.01
	0.05	0.1	3251	15,826	225	0.008	0.021
	0.05	0.2	3185	12,697	420	0.048	0.063
	0.1	0.05	3188	13,282	366	0.003	0.071
	0.1	0.1	3198	11,707	367	0.019	0.067
	0.1	0.2	2770	9076	792	0.167	0.41
	0.2	0.05	3008	8935	659	0.008	0.194
	0.2	0.1	2963	7630	707	0.008	0.222
	0.2	0.2	2789	5539	845	0.081	0.341
1.15$\tau_{0}$	0.05	0.05	3108	7768	500	0.012	0.154
	0.05	0.1	3010	6965	633	0.043	0.202
	0.05	0.2	2895	5588	785	0.109	0.261
	0.1	0.05	2874	5845	779	0.014	0.287
	0.1	0.1	2776	5152	854	0.041	0.345
	0.1	0.2	2364	3994	1069	0.126	0.533
	0.2	0.05	2381	3932	1112	0.014	0.491
	0.2	0.1	2324	3358	1115	0.042	0.538
	0.2	0.2	1952	2438	1206	0.151	0.663
1.2$\tau_{0}$	0.05	0.05	2642	4326	944	0.023	0.417
	0.05	0.1	2573	3879	966	0.047	0.443
	0.05	0.2	2358	3112	1085	0.133	0.534
	0.1	0.05	2280	3256	1100	0.019	0.562
	0.1	0.1	2181	2870	1149	0.057	0.598
	0.1	0.2	1890	2225	1165	0.14	0.713
	0.2	0.05	1531	2190	1155	0.027	0.809
	0.2	0.1	1574	1870	1188	0.073	0.798
	0.2	0.2	1231	1358	1111	0.166	0.874
1.3$\tau_{0}$	0.05	0.05	1635	1885	1149	0.042	0.804
	0.05	0.1	1436	1690	1116	0.094	0.846
	0.05	0.2	1215	1356	1033	0.15	0.909
	0.1	0.05	1208	1418	1099	0.032	0.881
	0.1	0.1	1110	1250	1039	0.087	0.912
	0.1	0.2	1053	969	1121	0.158	0.859
	0.2	0.05	791	954	933	0.056	0.951
	0.2	0.1	710	815	830	0.098	0.97
	0.2	0.2	502	591	645	0.176	0.988

**Table 7:** Sequential test results. Mixture of distributions Gamma p=0.1, $\tau_{0}=50$.

$\tau_{1}$	α	β	$\bar{n}$	$S(\bar{n})$	$\hat{\beta }$	$\hat{\kappa }$
1.01$\tau_{0}$	0.05	0.05	3067	642	0.067	0.139
	0.05	0.1	3044	655	0.094	0.164
	0.05	0.2	2978	690	0.134	0.224
	0.1	0.05	3047	662	0.071	0.151
	0.1	0.1	3022	672	0.076	0.172
	0.1	0.2	2972	731	0.121	0.215
	0.2	0.05	2983	733	0.075	0.191
	0.2	0.1	3005	673	0.082	0.195
	0.2	0.2	2927	751	0.126	0.255
1.03$\tau_{0}$	0.05	0.05	3010	713	0.08	0.165
	0.05	0.1	3033	678	0.091	0.168
	0.05	0.2	2974	708	0.135	0.215
	0.1	0.05	3014	674	0.089	0.179
	0.1	0.1	3010	696	0.083	0.183
	0.1	0.2	2951	740	0.136	0.226
	0.2	0.05	2956	713	0.095	0.232
	0.2	0.1	2963	733	0.095	0.229
	0.2	0.2	2895	792	0.11	0.249
1.05$\tau_{0}$	0.05	0.05	3000	717	0.079	0.176
	0.05	0.1	3019	701	0.108	0.171
	0.05	0.2	2935	746	0.155	0.241
	0.1	0.05	3007	699	0.072	0.185
	0.1	0.1	2989	693	0.102	0.213
	0.1	0.2	2919	759	0.16	0.273
	0.2	0.05	2936	752	0.088	0.232
	0.2	0.1	2952	729	0.109	0.238
	0.2	0.2	2861	790	0.157	0.298
1.075$\tau_{0}$	0.05	0.05	3017	687	0.087	0.165
	0.05	0.1	2999	706	0.106	0.184
	0.05	0.2	2968	718	0.143	0.231
	0.1	0.05	2994	694	0.078	0.2
	0.1	0.1	2963	756	0.107	0.208
	0.1	0.2	2940	767	0.131	0.238
	0.2	0.05	2885	793	0.093	0.272
	0.2	0.1	2891	747	0.119	0.286
	0.2	0.2	2832	819	0.156	0.326

**Table 8:** Sequential test results. Mixture of distributions Gamma p=0.1, $\tau_{0}=50$.

$\tau_{1}$	α	β	$\bar{n}$	$S(\bar{n})$	$\hat{\beta }$	$\hat{\kappa }$
1.1$\tau_{0}$	0.05	0.05	3021	674	0.097	0.176
	0.05	0.1	3010	656	0.118	0.198
	0.05	0.2	2963	719	0.131	0.234
	0.1	0.05	2979	714	0.104	0.215
	0.1	0.1	2925	779	0.108	0.237
	0.1	0.2	2859	808	0.163	0.288
	0.2	0.05	2891	748	0.07	0.296
	0.2	0.1	2888	743	0.1	0.313
	0.2	0.2	2725	794	0.177	0.437
1.15$\tau_{0}$	0.05	0.05	2953	757	0.087	0.213
	0.05	0.1	2986	707	0.098	0.213
	0.05	0.2	2901	738	0.169	0.298
	0.1	0.05	2849	799	0.083	0.319
	0.1	0.1	2696	847	0.227	0.454
	0.1	0.2	2779	822	0.161	0.396
	0.2	0.05	2620	878	0.08	0.48
	0.2	0.1	2531	933	0.11	0.504
	0.2	0.2	2416	923	0.165	0.608
1.2$\tau_{0}$	0.05	0.05	2861	781	0.071	0.329
	0.05	0.1	2844	778	0.093	0.335
	0.05	0.2	2692	818	0.186	0.458
	0.1	0.05	2655	882	0.075	0.46
	0.1	0.1	2668	844	0.091	0.47
	0.1	0.2	2441	933	0.204	0.59
	0.2	0.05	2264	968	0.064	0.644
	0.2	0.1	2163	980	0.09	0.712
	0.2	0.2	1964	956	0.19	0.794
1.3$\tau_{0}$	0.05	0.05	2419	926	0.083	0.599
	0.05	0.1	2369	923	0.107	0.632
	0.05	0.2	2131	926	0.171	0.758
	0.1	0.05	2079	969	0.078	0.737
	0.1	0.1	2013	929	0.13	0.787
	0.1	0.2	1789	927	0.17	0.853
	0.2	0.05	1615	996	0.062	0.858
	0.2	0.1	1521	925	0.105	0.903
	0.2	0.2	1345	863	0.158	0.943

**Table 9:** Sequential test results. Mixture of distributions Gamma p=0.2, $\tau_{0}=50$.

$\tau_{1}$	α	β	$\bar{n}$	$S(\bar{n})$	$\hat{\beta }$	$\hat{\kappa }$
1.01$\tau_{0}$	0.05	0.05	3125	532	0.046	0.094
	0.05	0.1	3236	308	0.017	0.027
	0.05	0.2	3185	443	0.037	0.061
	0.1	0.05	3226	340	0.009	0.032
	0.1	0.1	3199	406	0.024	0.055
	0.1	0.2	3190	408	0.046	0.07
	0.2	0.05	3174	448	0.026	0.067
	0.2	0.1	3188	434	0.023	0.061
	0.2	0.2	3146	508	0.043	0.09
1.03$\tau_{0}$	0.05	0.05	3061	611	0.068	0.127
	0.05	0.1	3162	486	0.042	0.071
	0.05	0.2	3170	455	0.049	0.071
	0.1	0.05	3168	453	0.033	0.079
	0.1	0.1	3149	509	0.035	0.078
	0.1	0.2	3118	543	0.069	0.103
	0.2	0.05	3146	477	0.037	0.095
	0.2	0.1	3101	572	0.048	0.117
	0.2	0.2	3073	600	0.061	0.136
1.05$\tau_{0}$	0.05	0.05	3045	654	0.074	0.128
	0.05	0.1	3178	440	0.035	0.065
	0.05	0.2	3148	479	0.059	0.091
	0.1	0.05	3184	416	0.024	0.069
	0.1	0.1	3148	491	0.034	0.092
	0.1	0.2	3155	498	0.042	0.077
	0.2	0.05	3154	483	0.03	0.086
	0.2	0.1	3096	576	0.039	0.126
	0.2	0.2	3106	533	0.073	0.134
1.075$\tau_{0}$	0.05	0.05	3048	632	0.062	0.133
	0.05	0.1	3171	446	0.04	0.074
	0.05	0.2	3104	571	0.081	0.118
	0.1	0.05	3138	520	0.043	0.09
	0.1	0.1	3151	491	0.041	0.089
	0.1	0.2	3109	544	0.07	0.118
	0.2	0.05	3143	487	0.043	0.102
	0.2	0.1	3100	565	0.047	0.117
	0.2	0.2	2937	878	0.076	0.363

**Table 10:** Sequential test results. Mixture of distributions Gamma p=0.2, $\tau_{0}=50$.

$\tau_{1}$	α	β	$\bar{n}$	$S(\bar{n})$	$\hat{\beta }$	$\hat{\kappa }$
1.1$\tau_{0}$	0.05	0.05	3032	662	0.087	0.143
	0.05	0.1	3150	513	0.044	0.077
	0.05	0.2	3137	511	0.062	0.093
	0.1	0.05	3156	487	0.034	0.085
	0.1	0.1	3122	538	0.048	0.105
	0.1	0.2	3095	582	0.067	0.122
	0.2	0.05	3119	527	0.034	0.116
	0.2	0.1	3066	606	0.048	0.142
	0.2	0.2	3012	655	0.086	0.197
1.15$\tau_{0}$	0.05	0.05	3154	480	0.032	0.09
	0.05	0.1	3139	510	0.05	0.095
	0.05	0.2	3095	565	0.089	0.133
	0.1	0.05	3130	515	0.035	0.109
	0.1	0.1	3264	175	0.047	0.012
	0.1	0.2	3061	598	0.07	0.163
	0.2	0.05	2987	648	0.035	0.237
	0.2	0.1	2985	655	0.043	0.242
	0.2	0.2	2885	710	0.09	0.317
1.2$\tau_{0}$	0.05	0.05	3134	529	0.034	0.103
	0.05	0.1	3102	552	0.047	0.129
	0.05	0.2	3063	567	0.094	0.195
	0.1	0.05	3029	603	0.035	0.195
	0.1	0.1	3043	592	0.049	0.208
	0.1	0.2	2986	638	0.115	0.407
	0.2	0.05	2773	800	0.035	0.379
	0.2	0.1	2726	809	0.055	0.429
	0.2	0.2	2578	874	0.131	0.507
1.3$\tau_{0}$	0.05	0.05	2954	639	0.036	0.299
	0.05	0.1	2881	699	0.064	0.339
	0.05	0.2	2742	799	0.138	0.440
	0.1	0.05	3033	611	0.038	0.206
	0.1	0.1	2595	858	0.069	0.513
	0.1	0.2	2318	938	0.164	0.641
	0.2	0.05	2172	991	0.039	0.674
	0.2	0.1	2106	993	0.074	0.712
	0.2	0.2	1848	995	0.168	0.801

**Table 11:** Sequential test results. Mixture of distributions Gamma p=0.3, $\tau_{0}=50$.

$\tau_{1}$	α	β	$\bar{n}$	$S(\bar{n})$	$\hat{\beta }$	$\hat{\kappa }$
1.01$\tau_{0}$	0.05	0.05	3249	241	0.014	0.025
	0.05	0.1	3244	238	0.022	0.036
	0.05	0.2	3243	244	0.025	0.037
	0.1	0.05	3245	235	0.016	0.032
	0.1	0.1	3256	185	0.017	0.032
	0.1	0.2	3235	242	0.029	0.05
	0.2	0.05	3238	244	0.017	0.044
	0.2	0.1	3236	254	0.024	0.048
	0.2	0.2	3235	238	0.031	0.055
1.03$\tau_{0}$	0.05	0.05	3240	267	0.016	0.032
	0.05	0.1	3233	245	0.023	0.042
	0.05	0.2	3204	329	0.049	0.076
	0.1	0.05	3230	256	0.025	0.048
	0.1	0.1	3222	277	0.031	0.057
	0.1	0.2	3193	316	0.057	0.085
	0.2	0.05	3212	309	0.023	0.068
	0.2	0.1	3205	328	0.037	0.079
	0.2	0.2	3187	361	0.05	0.095
1.05$\tau_{0}$	0.05	0.05	3241	225	0.026	0.043
	0.05	0.1	3225	269	0.032	0.058
	0.05	0.2	3208	326	0.048	0.064
	0.1	0.05	3222	284	0.02	0.056
	0.1	0.1	3216	288	0.032	0.068
	0.1	0.2	3216	275	0.053	0.08
	0.2	0.05	3205	328	0.03	0.074
	0.2	0.1	3212	302	0.031	0.07
	0.2	0.2	3180	371	0.045	0.097
1.075$\tau_{0}$	0.05	0.05	3233	253	0.022	0.048
	0.05	0.1	3209	335	0.039	0.06
	0.05	0.2	3203	330	0.048	0.072
	0.1	0.05	3217	296	0.021	0.062
	0.1	0.1	3211	303	0.035	0.067
	0.1	0.2	3190	349	0.053	0.09
	0.2	0.05	3206	321	0.023	0.075
	0.2	0.1	3204	317	0.035	0.08
	0.2	0.2	3178	355	0.065	0.106

**Table 12:** Sequential test results. Mixture of distributions Gamma p=0.3, $\tau_{0}=50$.

$\tau_{1}$	α	β	$\bar{n}$	$S(\bar{n})$	$\hat{\beta }$	$\hat{\kappa }$
1.1$\tau_{0}$	0.05	0.05	3232	263	0.027	0.044
	0.05	0.1	3220	260	0.042	0.064
	0.05	0.2	3189	345	0.052	0.086
	0.1	0.05	3214	323	0.029	0.054
	0.1	0.1	3199	356	0.032	0.066
	0.1	0.2	3193	339	0.064	0.091
	0.2	0.05	3179	389	0.025	0.087
	0.2	0.1	3182	364	0.004	0.095
	0.2	0.2	3142	434	0.076	0.132
1.15$\tau_{0}$	0.05	0.05	3222	276	0.033	0.054
	0.05	0.1	3213	308	0.036	0.059
	0.05	0.2	3169	384	0.074	0.102
	0.1	0.05	3197	338	0.036	0.08
	0.1	0.1	3197	338	0.04	0.074
	0.1	0.2	3197	296	0.049	0.01
	0.2	0.05	3163	376	0.032	0.124
	0.2	0.1	3162	380	0.043	0.123
	0.2	0.2	3111	460	0.063	0.179
1.2$\tau_{0}$	0.05	0.05	3214	299	0.029	0.056
	0.05	0.1	3201	322	0.046	0.076
	0.05	0.2	3154	397	0.085	0.119
	0.1	0.05	3190	335	0.026	0.099
	0.1	0.1	3171	365	0.051	0.123
	0.1	0.2	3085	486	0.092	0.19
	0.2	0.05	3014	552	0.037	0.248
	0.2	0.1	3002	565	0.05	0.263
	0.2	0.2	2924	594	0.098	0.347
1.3$\tau_{0}$	0.05	0.05	3121	421	0.036	0.17
	0.05	0.1	3087	484	0.054	0.198
	0.05	0.2	2997	578	0.121	0.271
	0.1	0.05	2954	560	0.031	0.31
	0.1	0.1	2935	598	0.061	0.327
	0.1	0.2	2775	746	0.106	0.415
	0.2	0.05	2612	839	0.03	0.504
	0.2	0.1	2450	865	0.05	0.602
	0.2	0.2	2280	928	0.134	0.673

## Data Availability

Data are contained within the article.

## References

[1] Kaplan R.S. (1973). A Stochastic Model For Auditing. J. Account. Res..

[2] Stringer K.W. Practical Aspects of Statistical Auditing. Proceedings of the Business and Economic Statistics Section of the American Statistical Association.

[3] Wywiał J.L. (2018). Application of Two Gamma Distributions Mixture to Financial Auditing. Sankhya B.

[4] Frost P.A., Tamura H. (1986). Accuracy of Auxiliary Information Interval Estimation in Statistical Auditing. J. Account. Res..

[5] Tamura H. (1988). Estimation of rare errors using judgement. Biometrika.

[6] Fisz M. (1967). Elements of sequential analysis. Probability and Mathematical Statistics.

[7] Wald A. (1945). Sequential Tests of Statistical Hypotheses. Ann. Math. Stat..

[8] Byekwaso S. (1994). Bayesian Sequential Inference for Error Rates and Error Amounts in Accounting Data. Ph.D. Thesis.

[9] Gölz M., Fauss M., Zoubir A. A bootstrapped sequential probability ratio test for signal processing applications. Proceedings of the 2017 IEEE 7th International Workshop on Computational Advances in Multi-Sensor Adaptive Processing (CAMSAP).

[10] Marek T., Noworol C. (1989). Wprowadzenie do Analizy Sekwencyjnej.

[11] Boiroju N.K. (2013). A study on bootstrap sequential probability ratio test. Proceedings of the VI International Symposium on Optimization and Statistics.

